# Animals in Moral Limbo: How Literary Pigs May Help Lab-Generated Ones

**DOI:** 10.3390/ani10040629

**Published:** 2020-04-06

**Authors:** Nancy Tuck

**Affiliations:** Albany Medical College, Alden March Bioethics Institute, 43 New Scotland Avenue, Albany, NY 12208, USA; nancytuck7@gmail.com

**Keywords:** animal welfare, animal moral status, animal experimentation, animal ethics

## Abstract

**Simple Summary:**

Unlike dogs and horses, who maintain “companion animal” moral status and protections from societal harms, pigs are increasingly being used in medical experimentation for human use, in spite of scientific validation of advanced porcine cognitive and social-emotional abilities (on a par with, if not greater than, other protected mammals). While animal ethicists apply philosophical paradigms in risk-benefit deliberations, this article challenges assumptions about societal/cultural norms of pig commodification and experimentation through application of a literary lens—specifically, how children’s coming-of-age stories have demonstrated increased pig moral status vis-à-vis human–nonhuman animal relationships and understandings. Human society must revisit pronouncements of “having no other choice” when it comes to increased promotion and continued acceptance, of medical experimentation, and industrial commodification of pigs. Xenotransplantation trials are especially morally fraught due to the purposeful generation of human–nonhuman chimeras in efforts to produce human organs for transplantation.

**Abstract:**

When considering that artistic and literary artifacts reflect the cultural views and mores of a particular time period, there is a significant misalignment between stories depicting increased moral status of pigs (e.g., vis-à-vis human-porcine relationships) and ongoing practices of pig consumption, commodification, and medical experimentation. In fact, there has been increased industrial farm meat production and biotechnological experimentation. Xenotransplantation trials, for example, are being heralded “the answer” to organ shortages needed for human transplantation, while significant ethical concerns persist. In this paper, I posit that literary reflections add a valuable dimension to animal ethics deliberations, providing a meta-narrative against which to assess normative practices. Beginning with synopses of three books: E.B. White’s *Charlotte’s Web* (1952), Robert Newton Peck’s *A Day No Pigs Would Die* (1972), and Paul Griffin’s *Saving Marty* (2017), I illustrate a shifting moral status view of human–pig relationships. Next, I discuss personhood attributions through biological, philosophical, and legal frameworks; review benefits and risks of xenotransplantation; reflect on the moral status of non-human animals; and offer concluding thoughts.

## 1. Introduction

It is often suggested that children’s stories are written for an adult audience; under the guise of childhood fantasy and innocence, these stories challenge readers to confront their own roles in perpetuating accepted practices and behavioral norms. From the earliest traditions of oral storytelling, through to today, animals have been featured prominently in folk tales, parables, myths, and origin stories. Specific to time period, geographic location, and cultural influence, animal portrayals have ranged from revered and beloved to reviled and dismissed. Monotheistic religions relegate animals to positions of human subservience in a hierarchical Great Chain of Being, a diminishment of moral standing viewed by some as a means of alleviating human guilt over animal ownership, commodification, or consumption. In mid-eighteenth century Western children’s literature, animal protagonists were assigned human voice, appearance, and behaviors; anthropocentric practices that have been criticized by many as speciest, perpetuating views of human superiority. Over time, animal voice or point-of-view has shifted from human-centric to animal-centric, a shift helping to spotlight issues of animal agency and personhood, the value of interspecies relationships, and factors driving conformance with societal norms.

While the majority of children’s stories feature domesticated companion animals, such as dogs and horses, stories featuring pigs as companion animals are particularly morally fraught for reasons that include: 1) religions casting pigs as dirty or ritually unclean, and 2) a deep-seated emotional unease resulting from interactions with animals who present as highly intelligent and social, yet are denied any of the societal protections and moral standing afforded other animals (e.g., aggressive breeding, confinement, and other questionable practices in the service of human consumption and commodification). Medical research also challenges the animal moral status continuum when pigs are used in clinical and genetic trials; xenotransplantation trials, for example, are being heralded “the answer” to organ shortages in human transplantation, while serious ethical concerns persist. Xenotransplantation is “the transplantation, implantation, or infusion into a human recipient of…live cells, tissues, or organs from a non-human animal source or human body fluids, cells, tissues or organs that have had ex vivo contact with live nonhuman animal cells, tissues, or organs” [1].

Bioethicists and animal ethicists are devoted to upholding the ethical treatment and protection of their respective human and non-human animal populations. With differing paradigms—animal ethics framed via orientations ranging from agency-based, to contextual, relational, ecological, or rights theory-based, and bioethics via orientations as diverse as principlist, feminist, virtue-based, egalitarian, deontological, utilitarian, communitarian, and more—and no consensus as to which framework(s) should predominate, interspecies ethics discussions are all the more daunting. In this paper, I suggest that literary reflections can add a valuable dimension to bioethics and animal ethics deliberations, providing a meta-narrative against which to assess normative practices. After decades of resistance by the science community, the soft sciences (e.g., psychology, sociology) are finally granted seating at the same deliberative table. And it is within this spirit of collaborative discourse that I suggest application of a literary lens to moral deliberations. Beginning with synopses and excerpts from three books highlighting a shifting moral status view of human–pig relationships, I review personhood attributions through biological, philosophical, and legal frameworks; discuss benefits/risks of xenotransplantation; reflect on moral status of non-human animals; and offer concluding thoughts.

## 2. Children’s Literature

When reviewing literary relationships between humans and pigs, there has been a noticeable moral paradigm shift over the past seventy or so years. In Western literary works written during the mid- and late-twentieth century, fictional human protagonists engage in mental rationalization processes justifying a “need” to diminish the moral status of their porcine companions, thereby changing their relationship status from one of reciprocal friendship and caring, to one of human utility. But over the past couple of decades, literary works featuring human–pig relationships depict human protagonists who continue to value their relationships, advocating for the ongoing physical and emotional well-being of their animal companions. Animal characterizations have also changed considerably. Pig protagonists are no longer in anthropomorphized form; rather, they maintain their own biological characteristics and are depicted via a relational framework.

When considering that artistic and literary artifacts reflect the cultural views and mores of a particular time period, there is a significant misalignment between stories depicting increased moral status of pigs (e.g., vis-à-vis human-porcine relationships) and ongoing practices of pig consumption, commodification, and medical experimentation. In demonstrating how literary moral paradigms of human–pig relationships have evolved, I offer analyses of E.B. White’s *Charlotte’s Web* (1952), Robert Newton Peck’s *A Day No Pigs Would Die* (1972), and Paul Griffin’s *Saving Marty* (2017).

### 2.1. Charlotte’s Web

In the beginning of E.B. White’s *Charlotte’s Web* (1952), 8-year-old Fern sees her father carrying an ax and heading to the barn to kill a pig, a runt of the litter who “will never amount to anything.” But Fern’s pleas, e.g., “If I had been very small at birth, would you have killed me?” [2] successfully move her father to forgo his mission, and allow Fern to keep the piglet, whom she names Wilbur. For the first few weeks, Fern dotes on Wilbur, attending to his every need. Yet when Fern’s father proclaims that Wilbur “is no longer a baby” and has to be sold to Fern’s Uncle, a farmer down the road, Fern’s response, “How much money should I ask for him?” denotes a ready acceptance of commodification seemingly incongruent with her earlier pleas. Fern is now satisfied in being allowed to visit Wilbur.

Fern’s visits soon decrease, leaving Wilbur sad and lonely, until he befriends the animals in the barn, forming a special bond with a spider named Charlotte. When Wilbur finds out that the farmer is fattening him up to kill him for food, he is horrified, but Charlotte quickly assures Wilbur that she will not let him die. Through a series of web-spun words that astound the farm’s owners, convincing them and all the visiting spectators of Wilbur’s specialness, his life is ultimately spared.

In an essay written by White in 1941, prior to *Charlotte’s Web*, he advises new writers, “Don’t write about Man; write about a man” [3]. This sentence underscores an important psychological factor; in writing about a specific pig, as Charlotte does (woven words into her web) in the story, rather than a categorical reference to pigs, she successfully secures the attention of the farmer and villagers. This emotional draw can be likened to news articles featuring a specific child or animal, with an identifiable face, name, and story, a “proximity effect” that invokes audience concern and compassion; these emotions, in turn, motivate actions to remedy the situation.

Fern’s relationship with Wilbur changes, as she is “growing up, and…careful to avoid childish things, like sitting on a milk stool near a pigpen” [2] “Growing up” is equated with Fern distancing herself from Wilbur, a negation of relationship that diminishes Wilbur’s moral status. No longer able to depend on Fern for love, comfort, or survival, Wilbur must instead rely on other animal species for empathy and protection, evidenced by his friend Charlotte’s statement, “Your success in the ring this morning was…my success. Your future is assured. You will live, secure and safe, Wilbur. Nothing can harm you now…you mean a great deal to Zuckerman and he will not harm you, ever” [2]. Farmer Zuckerman will not harm Wilbur because Wilbur has become important, not as a fellow being with moral worth, but as a commodity—a ticket to fame and money, rather than a meal ticket as originally intended. Charlotte understands that the only way to keep Wilbur alive is to find an alternate means of commodification, one that will satisfy his human owners.

Poignantly, a lack of human-to-pig altruism is underscored by Charlotte’s interspecies altruism, declaring, “Your success was my success.” And the deep emotional connection the two share is highlighted in Charlotte’s response to Wilbur’s lament that he cannot repay her kindness and heroism, “You have been my friend…That in itself is a tremendous thing” [2].

### 2.2. A Day No Pigs Would Die

Robert Newton Peck’s *A Day No Pigs Would Die* (1972) is the story of Rob, a 12-year-old boy from rural Vermont who grows up in the Shaker tradition. The Shakers, a Quakers sect, live by a strict code of prayer, work, communalism, and non-materialism. Family farming is a way of life, one that is self-sustaining. The animals all serve a specific purpose to the humans who own them, from providing milk, eggs, and wool, to meat food provision, to being sold to outside markets for income. In this story, Rob’s father is known for his pig butchering skills.

The book starts out describing Rob’s rescue of a neighbor’s cow, whom he stumbles upon in a field birthing a calf and in great distress, as the calf she is trying to deliver is stuck. Rob pulls the calf out, and then dislodges a “goiter” from the cow’s throat to open up her airway; these brave actions save the lives of both cow and calf but leave Rob with significant injuries. The grateful neighbor brings Rob a thank-you gift of a newborn piglet, a gift Rob’s father is initially reluctant to let him accept, since neighbors are supposed to look out for each other and each other’s animals. But when the neighbor reframes the offer as a belated birthday gift to Rob, his father acquiesces, and a thrilled Rob embraces his piglet, naming her Pinky. “’Pinky’s a fitting name,’ said Mama. ‘Never heard of naming a pig,’ Aunt Carrie said” [4].

Unlike *Charlotte’s Web*, *A Day No Pigs Would Die* was banned by many libraries and schools across the nation during the 1970s and 1980s, due to violent and graphic portrayals deemed inappropriate for a children’s audience, e.g., a dog and weasel fighting to the death in a closed barrel (when the dog’s owner is “training” the dog to hunt weasels), or a farmer’s attempt to mate his boar with a barren pig, convinced this will “fix” her “estrus problem” (a violent mating scene resulting in serious injuries to the pig). Of note, however, the graphic slaughtering of pigs *was not* among the reasons given for library and school bans of the book [5].

Rob reflects on his father’s work; “I could tell by the smell of his hand that he’d killed pigs today. There was a strong smell to it, like stale death. That smell was almost always on him…He smelled best on Sunday morning, when I sat next to him at Shaker Meeting” [4]. Rob takes his schooling and farm chores seriously, but his favorite task is caring for Pinky. Intrigued by Pinky’s playfulness and intelligence, he enjoys their time together and takes great comfort in her affections (e.g., her enthusiastic greetings and physical nudges). “It always looked to me like she was smiling…just like I could smile to see Pinky, she sure could smile to see me” [4]. Rob loves and is protective of Pinky, stating, “And I’m going to keep right on taking care of you proper…You ain’t going to be pork. No missy” [4].

As the story progresses, Rob’s family is confronted by a harsh winter, poor crop harvest, lack of hunting success, and no remaining piglets nor other animal offspring to sell at market. And, to make matters worse, Rob’s father confides that he is has been ill and not sure he will make it to the spring season. Before contending with the loss of his father, however, Rob is forced to deal with the heartbreaking loss of his beloved Pinky, as Rob’s father utters the pronouncement, “Help me boy…It’s time” [4], indicating his father’s decision to kill Pinky for meat to feed the family. Adding to the shock of this announcement, Rob is told he must assist in the slaughter as proof he is “man” enough to provide for his family when his father is gone.

The reader shares Rob’s anguish when he approaches the barn, and Pinky excitedly rushes to him. “She came to me, nuzzle pointed into my leg. Her curly tail was moving about like it was glad the day had started. People say pigs don’t feel. And that they don’t wag their tails. All I know is that Pinky sure knew who I was and her tail did too” [4]. Rob says to his father, “I don’t think I can”, to which his father responds, “That ain’t the issue, Rob. We have to” [4].

Rob has a mental dialogue with Pinky, rationalizing a necessity for what is about to happen to her. “Pinky…try and understand. If there was any other way. If only Papa had got a deer this fall. Or if I was old enough to earn money. If only…” [4].

After a graphic portrayal of the father’s slaughter of Pinky, with Rob’s assistance, Rob’s inner dialogue is one of fury; “I hated Papa that moment. I hated him for killing her, and hated him for every pig he ever killed in his lifetime” [4]. His father finishes butchering Pinky and Rob is filled with grief, “’Oh, Papa. My heart’s broke.’ ‘So is mine,’ said Papa. ‘But I’m thankful you’re a man’” [4].

When Rob’s father dies a few months later, Rob must assume his position as head of the family; at only thirteen years of age, his first job is arranging his father’s funeral. Rob solemnly prepares to make a eulogy, and lead the procession and burial, knowing everyone in the community will be putting on their Sunday best to attend the burial of his father. “There would be no work on this day. A day no pigs would die” [4]. The story leaves off with images of Rob’s stoic acceptance of his responsibilities, a final tamping down of any emotion (or possibilities of healing). “There wasn’t much to eat, except beans…we’d lived on those all winter, beans and pork. And none of it was easy to swallow” [4].

In *A Day No Pigs Would Die*, Peck masterfully portrays a nexus of conflicting trajectories: an adolescent’s coming-of-age while growing up in a strict tradition of observance and expected behavior (a set of norms nested within the cultural norms of a larger society); a child’s direct experiences of relationship-building with an animal exhibiting sophisticated cognitive and affective abilities, thus intuiting animal moral status that is glaringly misaligned with a view of animal consumption; a child with conflicted emotions—one who admires and strives to emulate his father, while simultaneously despising the nature of his father’s work; an untimely parental loss that forces a child into adulthood (according to an imposed definition of adulthood), and the trauma of a child being made to directly participate in killing the animal he loves, and no emotional recourse for healing. 

A helpful perspective is offered by Gary Comstock, a Mennonite from Ohio who grows up in a family farming tradition, wherein pigs and livestock are well cared for until they are sent off to slaughter. Comstock discloses he has made a conscious decision of vegetarianism, thus rejecting norms of meat-eating, while also yearning to maintain familial and cultural acceptance. Embarking upon a spiritual and emotional journey, Comstock struggles to make sense of conflicting Biblical messages regarding man’s relationship with animals and searches for a philosophical paradigm that can align with his intuitive moral instincts. “I did not have a hard time deciding whether pigs experience pleasure and pain, or whether they have emotions, desires, wishes, preferences, and a family life. All of this seemed evident to me from watching the pigs on my Uncle’s farm” [6].

Comstock comes to the realization that “facts alone, no matter how many, would never add up to the moral judgment that it is wrong to kill and eat *Sus scrofa domestica”* [6]. He discusses the vastly differing perspectives within the ethics community; some posit that in order to have moral rights, “something must be conscious, capable of taking an interest or able to have an interest in what is good for it” [6], while others advocate equal moral status for animals as for humans. There is also a bifurcated view among ethicists, differentiating between moral status of *wild* versus *domesticated* animals; some believing that humans can ethically utilize domesticated animals to meet human needs, including killing them if done “humanely”, while others believing that once we interfere in nature’s course through domestication of animals, we have an increased obligation to protect the animals we have made dependent upon us. Comstock questions: Do wild pigs and domesticated pigs have different moral worth? Do wild pigs have intrinsic value, while domesticated ones only instrumental value? Does the manner in which pigs are killed (e.g., “humanely”) lessen the wrongness of killing them?

### 2.3. Saving Marty

In Paul Griffin’s *Saving Marty* (2017), we learn that 11-year-old Renzo’s father, an army medic and decorated war hero, died a week before Renzo was born. Renzo’s family (consisting of Renzo, his mother, and grandfather) can barely make ends meet, living in a poor rural area, at the foot of an orchard no longer producing sellable fruit. They have a few animals, but they are mostly for making money, e.g., breeding and selling puppies and piglets. One day, when Renzo goes to the barn to visit the litter of puppies just delivered by his beloved dog, Bella, he discovers a piglet that was left behind, a runt of a litter that was just sold. Bella treats the piglet like one of her own pups, nursing and protecting him in a maternal act of interspecies altruism. Before long Renzo’s mother finds out about the piglet, stating, “You know that’s livestock, right? You know you’re not keeping him” [7]. Renzo pleads with her to let him keep the piglet, and she reluctantly acquiesces; he can keep him for a few weeks. Renzo decides on a name, *Marty*, his father’s name. Names are important because, as Renzo informs his friend Pal, “when you name somebody, he stays with you forever” [7].

While Renzo and Marty develop a strong bond, Renzo’s mother has no patience for a pig who figures out how to get into the icebox and eat their food. Desperate to figure out a way to save Marty from auction, Renzo enters Marty into a community dog race that offers a cash first prize. Marty is a pig who thinks he’s a dog, after all, and the judges admit that there are no rules against entering a pig. But Marty’s victory angers a competing dog owner whose own dog was edged out of the win; a man who threatens to shoot Marty as pay-back. Not only must Renzo be on alert for Marty’s physical safety, but Marty keeps getting bigger and bigger, making it harder for Renzo to keep him out of trouble (e.g., rooting up the neighbor’s yard).

Renzo’s mother has had enough, but realizing how attached Renzo has become to Marty, she cannot bring herself to sell Marty at auction. The animal sanctuaries are at their limit, but she is able to find a petting zoo willing to take Marty. When Renzo hears of her plan, he forces himself to nod in acceptance, “but there was no chance I was letting Marty go to a zoo up in Michigan… He would forever wonder what he’d done to make me send him away” [7].

Renzo thinks there is more to the story of his father’s death than his mother and grandfather are not letting on; upon voicing a theory to his mother that maybe his father is not dead but married someone overseas, his mother breaks down, telling Renzo the secret she has kept from him all this time—that his father shot himself, suffering from Post Traumatic Stress Disorder (PTSD). Reading the final letter his father had written, Renzo is shocked, angry, and heartbroken; he needs a way to process the pain. A pain and void that only Marty can fill; “that pig wanted nothing more than to love me and have me love him back. The worse things got, the more he was there for me” [7].

Determined not to let Marty be sent to a zoo, with his friend Pal accompanying him upon a long, dangerous trek to get Marty to an animal sanctuary, Renzo is attacked by a guard dog. Marty fights the dog off Renzo, sustaining serious injuries in the process. Informed by the sanctuary’s veterinarian there is a good chance Marty will not make it, Renzo is devastated by the news, “He was my teacher, I wanted to tell her. He taught me that the chance to look after somebody is a gift…the kind where you’d risk your lives to take care of each other… I whispered into his ear, ‘You’re my hero, Marty. Thank you for saving me’” [7].

The term “hero” resurfaces throughout the story. Griffin challenges readers, through Renzo’s reflections and struggles, to revisit this term in all of its ramifications, e.g., How much will we sacrifice to help others? Who are these “others” we care about? When do we challenge societal norms? Who helps us to grow, heal, and be whole? Do we live according to our true values? As readers ponder these questions, the story concludes on a hopeful note. Marty survives and is accepted to the animal sanctuary. Renzo volunteers at the sanctuary, where he gets to visit Marty and care for the other rescued animals. And Renzo will follow in his father’s footsteps to become a medic, a mission of healing that is a fitting tribute to his father and porcine brother.

On the surface, the above three novels serve as *Bildungsroman*, or tales of psychological and emotional growth signaling a character’s transition from childhood to maturity. Beyond issues of whether to accept or reject norms of society-at-large, the child protagonists in these stories jeopardize continued acceptance within their immediate families if they do not follow expectations. In processing each of the above stories, especially the first two, readers are asked to reflect upon more than human coming-of-age trials; not only do “these transformative learning experiences (consist of) relationships that a central character must choose to continue or terminate” [5], but the animal protagonists, as essential characters themselves, are literally caught between life and death. Whereas a terminated human-to-human relationship will result in emotional angst, a terminated human-to-animal relationship will result in the animal’s demise. Even in situations where animals are assigned moral worth as companion animals (e.g., dogs), long-term ethnographic research reveals that many loving relationships with animals do not endure; “when life changes and unexpected situations pose obstacles to the human–animal love, the people involved may redefine or terminate it. Pets are treated as ‘flexible persons’ or ‘emotional commodities’…(they) can at any moment be demoted and moved outside of the home and the family” [8].

We have learned a great deal over the past few decades about psycho-physiological maturation processes via advances in medical, technological, and social sciences. Some of these insights include: a greater appreciation of adolescent development and psychosocial struggles; increased recognition of, and treatment options for, people suffering through loss/grief and PTSD; more nuanced understanding of human and animal neurological processes (e.g., via functional magnetic resonance imaging (fMRI)) brain scan technologies); an appreciation for sophisticated communication systems within, and across, animal species (not just mammals); and evidence of animal sentience, advanced cognitive, and social abilities (more included in the upcoming section discussing biological personhood).

When Renzo’s mother, in *Saving Marty*, decides they can no longer keep Marty at home, she is sensitive to the value of the relationship between Renzo and Marty (a validation of relational moral status), and no longer contemplates selling Marty; the only acceptable options are to place him in an animal sanctuary or a zoo, where Marty will live a quality of life befitting an animal holding moral status. Even if Marty had not ended up in Renzo’s preferred animal sanctuary environment, he at least would have been allowed to live out his life in a protected environment (now required because domestication precludes release into the wild).

Just as in White’s *Charlotte’s Web* and Peck’s *A Day No Pigs Would Die*, relationships between people and pigs written before the turn of the century, “unfold as tragedies of sacrifice and betrayal, a storyline that tests an interspecies morality framing domestication as ‘animal consent’” [5]. A feminist lens contends that a domestication covenant upholds a “masculinized ethic of exploitation, valorizing the expectations of humans by presuming non-human ‘consent’ as a justification for violence and self-gratification…positioning Nature as a feminized ‘other’ to be conquered…this worldview trivializes and dismisses the affective and aesthetic ways of knowing nonhuman nature” [5].

By refusing to terminate the relationship as it gets harder to maintain, as exemplified by Renzo in *Saving Marty*, young protagonists “disrupt anthropocentric norms and incline to a biocentric worldview anchored in relational understandings of the other-than-human world. In honoring improbable interspecies friendships despite social and economic pressures to abandon them, they come of age by refusing to regard a non-human life as disposable” [5].

## 3. Personhood 

### 3.1. Biological Personhood

Pigs share several key similarities with humans in body size, anatomical features, and physiology. Plus, “as food animals, there is wide public acceptance of their…use, which is not the case for other nonrodent species, such as primates” [9]. This section addresses the many ways that pigs have been used in medicine and directions that biotechnological experimentation is heading.

In the 1960s, scientists began experimenting with pig xenograft (animal source grafts) aortic valve transplantations in humans. Although these early years met with significant failure due to transplant rejection, significant progress has since been made due to development of anti-rejection medication protocols and strategies to address structural valve deterioration [10]. Risk factors have diminished, yet challenges persist. 

Skin allografts (tissue transplants composed of different genotype within the same species), also known as allogeneic graft and homograft, are the gold standard for temporary skin coverage for burns. Allografts provide a viable barrier in the early phase after a severe burn but will be rejected if left too long and must eventually be replaced with an autograft (patient’s own skin tissue) [11].“Fresh, cryopreserved, or glutaraldehyde-preserved pig skin has been used widely as a temporary dressing for burns…Pig skin is similar to human skin in its histologic structure, and is an inexpensive and readily-available source” [11].

Cardiovascular diseases (CVDs) are the most common cause of human morbidity and death. Pigs are “suited to model human CVDs because of similarities in their cardio- and cerebrovascular systems, blood parameters and vessel size”; they are also susceptible to “atherosclerosis that can be accelerated by an atherogenic (high fat) diet” [9].

Cancers are the second leading cause of death, with numbers increasing as human populations age. “Porcine oncology is a new field and the extent to which pigs replicate human cancers will become clearer as more models are characterised” [9] In colorectal cancer (CRC) research, scientists posit “that pigs are perhaps more suitable than mice [and]…have generated pigs that …develop polyps in the colon and rectum as early as 4 months of age” [9]. To model diabetes, pigs have been generated to “express a human dominant-negative GIP receptor mutant (GIPRdn) in pancreatic islets” [9]. Scientists are also studying pigs with a “humanised immune system…Pigs engineered in this way could provide information about the response of the human immune system to cancers, infections and grafts” [9].

Due to similarities between porcine and human brains, pig brains are being used in the study of neurodegenerative disorders. Scientists have generated pig models that recapitulate early stage human Alzheimer’s Disease, confirming that porcine brain biology is similar to human [9]. In addition to Alzheimer’s, other neurodegenerative diseases being researched through pig disease modeling include Parkinson’s, Cystic Fibrosis, and Duchenne Muscular Dystrophy.

Turning to discussion of pigs’ cognitive abilities and socialization skills, there is significant evidence attesting to sophisticated psycho-social abilities, with pigs outperforming dogs on some tasks, and performing on a par with chimpanzees on others. Research scientists Marino and Colvin (2016) offer the following list of advanced porcine abilities:

**Memory**: pigs have sophisticated abilities to distinguish objects in a range of situations that require robust memory; **Symbolic language comprehension**: pigs understand gestures and verbal symbols that represent objects, as well as actions, and can learn complex combinations of symbols for actions and objects; **Time**: pigs detect the passage of time, remember specific events, and anticipate the future, e.g., “pigs could choose between two crates, each of which they had learned to associate with different lengths of confinement. Pigs also… anticipate whether positive or negative experiences might be imminent” [12]; **Functional flexibility**: pigs will utilize different methods to achieve a task, e.g., when they could not push a lever because their hooves kept slipping, they used their snouts to push the lever; **Problem-solving**: pigs “are whizzes with mazes and tests” and “outperform dogs in learning mazes [12]; **Numerosity**: experiments suggest that pigs possess an understanding of quantity; **Inventiveness**: pigs are inventive in their play, both with objects and with other pigs; **Social cognition and differentiation**: pigs can differentiate between members of another species, e.g., able to tell humans apart based on physical characteristics, smell, and voice; **Perspective-taking and_intentionality**: Pigs can take the perspective of other pigs and use this information to manipulate each other, and they sense the “attention state of humans” [12]; **Self-awareness**: researchers use the mirror self-recognition (MSR) test to determine if animals recognize themselves in a mirror. Pigs have been observed making repetitive movements while appearing to watch themselves in a mirror; this behavior is called contingency checking; **Cause and effect**: pigs understand that a joystick they control moves an on-screen cursor and outperformed dogs in manipulating a joystick to move a cursor to hit an on-screen target; **Focused attention**: Dr. Sarah Boysen, animal cognition researcher notes “pigs are capable of focusing their attention with even more intensity than a chimp” [12]; **Empathy**: pigs are sensitive; they “exhibit emotional contagion, a capacity thought to be the basis for empathy, or the ability to feel the emotional state of another…emotional experiences…are clearly evident in their play, fear and stress responses, and their sensitivity to the emotions of their companions” [12]; **Personality**: pigs display unique attributes and stable personality traits in “dimensions of aggression, sociability, and exploration. Such aspects of personality correlate closely to the human characteristics of agreeableness, extraversion, and openness” [12].

### 3.2. Philosophical Personhood

In Mycenaean Greece, the brave and ferocious wild boar was sacred to Ares, the god of war [13] In Europe and Asia, “gods were often associated with boars…wild pigs were the favorite animals of fertility gods” [13]. The Chinese consider the pig a symbol of both fertility and wealth and people “born in the Year of the Pig are believed to be blessed with the porcine qualities of sincerity, honesty, and kindness” [13].

After the transition from religious traditions of animism and polytheism to monotheism, pigs went from revered to reviled—prohibited, within Judaism and Islam, for religious use and consumption. Historians and anthropologists have postulated that these religious prohibitions were for sanitary, health related reasons (e.g., dangers of ingesting undercooked pork), and additionally, that monotheistic separation from polytheistic practices necessitated a complete disassociation from porcine imagery. Western philosophical perspectives, e.g., Aristotelian and Cartesian, paralleled Christian ones, wherein pigs were deemed devoid of moral status [14].

Today’s Western philosophical beliefs systems are influenced by European philosophers of the Enlightenment period (17th to 19th centuries). A paradigm often applied in bioethics discussions was first promoted by Immanuel Kant, a deontological, intention-based philosophical paradigm wherein an agent’s intent of action is said to matter in a given situation (as opposed to a focus on consequences, a utilitarianist paradigm). Kant promoted a categorical imperative; the rightness or wrongness of actions depends on fulfilling one’s duty as a rational agent, Although Kant’s emphasis on rational agents being afforded moral status (thus precluding infants or the cognitively or emotionally impaired) has drawn heavy criticism by many in the philosophical community, Kant believed it wrong to harm animals unnecessarily or treat them as disposable [15].

Charles Darwin, naturalist and biologist well-known for his theories of natural selection proposed in *On the Origin of Species* (1859), was a proponent of animal sentience and intelligence. Among his observations, Darwin wrote, “I have observed great sagacity in swine,” citing their intelligence to be at least, if not greater, than that of dogs [13]. Darwin disputed biological taxonomies, such as that developed by Carl Linnaeus, as arbitrary classification systems.

The field of bioethics (ethics of medical practice and experimentation) emerged as a discipline of due diligence after too many instances of unconscionable human experimentation (e.g., concentration camp victims in Nazi Germany during the holocaust or the Tuskegee Syphilis experiments in the United States, to name two examples). It was not until the latter part of the twentieth century that animal ethics began gaining traction, as well. The longstanding animal-welfare organization, the Society for the Prevention of Cruelty to Animals (SPCA), has been in existence since 1824, but the transition from animal-welfare to animal-rights advocacy did not occur until groups like People for the Ethical Treatment of Animals (PETA) was founded in 1980 [16].

Peter Singer, known for his utilitarian or consequentialist philosophical views, brought attention to the plight of animals, using the term “speciesism” to describe the discrimination encountered by animals due to human-imposed categories of species membership. Credited for exposing the inhumane factory farming practices of meat production, Singer has been a vocal critic against all forms of animal consumption, commodification, and experimentation. Singer advocates an expanded utilitarian paradigm—a premise promoting the greatest good for the greatest number and minimizing harms—as one that is inclusive of animals.

Ethics deliberations often center around discussions of justice. Animal ethicists have expanded upon a justice framework first proposed by John Rawls—a social contract theory rooted in the idea of justice as fairness (*A Theory of Justice*, 1971). According to Rawls, an agent must evaluate principles of justice from behind a “veil of ignorance” to eliminate any possibilities of bias. Animal ethicists have extended this thought experiment to include animals; when the veil is lifted, the beings encountered “may or may not be human, and they may or may not be rational…they (the agent) will be motivated to extend justice to everyone regardless of… species membership” [17].

In Martha Nussbaum’s view, pervasive power inequities (human-to-human and human-to-animal) compel us to “search for a different way to extend justice to animals. This different way…rooted in capacities…lends support to the view that nonhuman animals can be persons” [17]. In Nussbaum’s capabilities ethics approach, we all come to be persons through embeddedness in interpersonal relationships, shifting the “emphasis from individual traits and capacities to social relationships” [17].

Feminist care ethics, a perspective attributed to Carol Gilligan and encapsulated in her book, *A Different Voice* (1982), posits that views of morality often center on masculinized conceptions of justice, duties, or abstract concepts, rather than feminized conceptions of empathy, compassion, and care-taking. Ethicists applying a care ethics approach hold that humans must “learn to understand animals’ knowledges, languages, and communications…Care theory…is at base a dialogical ethic that entails listening to ‘different voices’ than the dominant” [18]. 

The concept of personhood is a contentiously debated topic, taking on differing philosophical meanings (and legal ones, discussed in the next section). Applying a “cluster” approach to personhood, “individuals must have some—and tend to have several—of the personhood traits, but no one of the traits is required” [17]. These include: Sentience, associated with basic awareness; Emotions, including happiness, empathy, sadness, fear, anger, or pain; Autonomy, the ability to act on behalf of oneself, exercising control over the formation of one’s goals and the means for achieving them; Self-awareness, of one’s own mental life; Sociality, in relation to other individuals; Language, used to communicate to others and self; Rationality, means-end reasoning or logical thought processes; Narrative self-constitution, thinking of oneself as a persisting subject with past experiences, a character in one’s own story who will author one’s future experiences; Morality, an understanding of what is good, right, or virtuous; Meaning-making, a vision of a life worth pursuing or a sense of what it is to live well.

Engagement in “normative practice” has also been cited as evidence of animal moral status; in primates, for example, family identities, group alliances, and in-group versus out-group practices offer a “mechanism for both delineating group identities and identifying out-group individuals, much in the way language, ritual, dress, etc., serve this purpose in human cultures” [19]. For decades, primatologists have observed and written about primates exhibiting empathy, reciprocity, conflict resolution, a sense of fairness, and cooperation [19]. Marine biologists have reported cetaceans exhibiting group identities, affiliations, dominance hierarchies, and unique preferences, e.g., a subgroup of dolphins in the “Shark Bay community use sponges as foraging tools, whereas other subgroups do not” [19].

Mammals and cetaceans not only exhibit remarkable intra-species social behaviors but evidence inter-species social behaviors, too. Whether through heroic actions, such as rescuing other animals or humans in danger, or self-sacrificial behaviors, such as engagement in mass strandings, “cetacean social practices exhibit norms of obedience, reciprocity, caring, social responsibility, and solidarity…(and) the cognitive capacity for normative ought-thought that is foundational to normative practice and to moral psychology” [19]. Ought-thought contemplations are sophisticated cognitions required for situational moral assessment and behavior. If “we remove the anthropocentric lens that has obscured some research, we can see that some claims of human uniqueness with regard to normative practice – and perhaps even the foundations of morality – may be spurious” [19].

### 3.3. Legal Personhood

Only recently, a few countries have begun to acknowledge animal rights, e.g., courts in Argentina and Colombia have granted habeas corpus to apes and a bear, and the Indian Supreme Court has recognized fundamental animal rights [20]. In the United States, judges have considered, but not endorsed animal rights, having denied the granting of “habeas corpus to chimpanzees and the standing of a macaque in a copyright suit” [20]. The Nonhuman Rights Project (NhRP), which has filed petitions on behalf of primates for habeas corpus (in attempts to allow their transfer to sanctuaries and away from conditions of extreme confinement), has argued a broad view of personhood–that an entity is a person if they can bear rights *or* responsibilities, and not necessarily both; a “reason for this broader interpretation is…not all persons can be held accountable for their actions and bear societal duties. Infants, children, and those found not guilty by reason of insanity cannot be held accountable and cannot bear societal duties. They are, nonetheless, persons with rights” [17].

Balancing the interests of animals against those of humans typically ends up prioritizing the latter. While “animals do not need free speech, freedom of religion…sentient animals would benefit from acknowledgment of a right to life, a right to be free from torture, and physical liberty” [20]. Animal ethicists have suggested that many of the same arguments that led to the codification of human rights in international agreements would be relevant for animal rights, as well. Rather than rely on domestic policies of fairness and justice, “such rights would belong to animals independent of their place of birth and abode…therefore universal…(and) international rights would serve as a benchmark for domestic law. International instruments would potentially allow for some monitoring…(or) criticism against domestic practices that do not satisfy the international standard” [20].

In legal contexts, personhood is important as a precondition for holding rights [20]. Yet, even in the eyes of the law, personhood is a “cluster concept that does not depend on a set of definite properties but has blurry boundaries…an actor or an entity can be a person for some purposes and a nonperson for others…international law is particularly open to the personhood of nonhumans” [20]. The concept of personhood, “with all its moral and legal weight, is not a biological concept…Any attempt to specify such a concept either leaves out a considerable number of humans—often the most vulnerable in our society—or includes members of other species” [17].

Animal rights activists argue that in applying principles of equality, “all sentient animals, independent of their mental capacities, must have the basic right to not be someone else’s property, just as all sentient humans, independent of their mental capacities, have the basic right to not be someone else’s property” [14]. According to current knowledge, all vertebrate animals (including fishes, birds, and mammals) are sentient to various degrees, and so are some advanced invertebrate animals [14]. Regardless of any differences in cognitive ability “there is no reason to believe that higher intelligence should be linked to a greater ability to feel emotions or pain…some emotions may even be experienced to a greater degree in some animals than in humans [14].

Informed consent in animal research is an issue that animal rights activists argue has not received the consideration needed. Within bioethics, research trials involving humans require fully informed consent; if an individual is unable to offer consent, there must be a surrogate/guardian appointed. But there is no equivalent “consenting” process with animals. For animals, consent is always given by others, and these “others” are not animal-appointed surrogates. Research trials involving animals require institutional review board approval, and must comply with federal, state, and international guidelines for “humane” treatment (e.g., physical pain management). Such regulatory bodies include: the Animal Welfare Act (1966); the Health Research Extension Act (1985), the Public Health Service Policy on Human Care and Use of Laboratory Animals, the Institutional Animal Care and Use Committee; international accreditation by the Association for Assessment and Accreditation of Laboratory Animal Care; and state-specific regulations.

Due to the wide regulatory gaps between animals used in food production and those used in research, some research scientists have utilized these gaps to their advantage by conducting their experiments on animals slated for food production, experiments that would not be approved under animal research guidelines. In a Nature article entitled “Part Revived Pig Brains Raise Slew of Ethical Quandaries” (2018), scientists experimented on “brains of pigs that had been decapitated for food production four hours before” [21]. The report continues, “Brain*Ex* study did not breach any ethical guidelines for research. The team sought guidance from Yale University’s Institutional Animal Care and Use Committee…(and) the committee decided that oversight was unnecessary. The pigs, having been raised as livestock, were exempt from animal welfare laws and were killed before the study started” [21]. This is a troubling research loophole; research scientists sidestepped federal regulations, garnered Institutional Review Board (IRB) approval, and lowered the protective bar, rather than upholding a higher ethical standard stipulated by animal research guidelines. It is also important to note that the “policies and regulations of the U.S. Public Health Service…do not specify any protections for animals after their death” [21]. These experiments only confirm the need to institute animal protections after death–in the course of the above study, scientists “restored and preserved some cellular activities and structures” [21] of the pig brains, casting further doubt on what constitutes death or lack of sentience in animals, making these ethical-work-arounds all the more troubling.

## 4. Xenotransplantation

Xenotransplantation is “the transplantation, implantation, or infusion into a human recipient of…live cells, tissues, or organs from a non-human animal source or human body fluids, cells, tissues or organs that have had ex vivo contact with live nonhuman animal cells, tissues, or organs” [1]. Reasons cited for pig xenotransplantation include “the similar size of pig organs to human organs; the ease with which it is possible to clone and genetically modify pigs; the large number of progeny; and the fact that pigs have a relatively short reproduction time and require only about 6 months to grow a sufficiently large and transplantable organ” [22].

To date, most patients with a porcine transplant need to take immunosuppressive medication, but “advances in stem cell and gene editing (CRISPR/Cas9) technologies now…bring the scientific community closer to developing human organs in a non-human animal…(by) seeding a pig blastocyst or embryo with human stem cells that would eventually grow into the desired organ” [22]. Gene editing would also be necessary to delete dozens of oncogenic porcine retroviruses that could trigger malignancies or zoonotic infections in the transplant recipients [22]. Researchers at the Salk Institute have already grown human tissue inside a pig embryo. A team at Emory University announced in 2017 that “a kidney from a genetically engineered pig was transplanted into a rhesus monkey and sustained that monkey for more than 400 days before being rejected” [22].

To prevent tissue rejection, a transplanted organ must contain at least 90% human cells, which “will make it necessary to generate a human organ with a human vascular system…we do not know how many pig chimeras will be needed to ensure that a proper patient-specific organ is obtained for transplantation” [22]. There is concern about a humanization effect, or “the possibility that non-directed human iPS cells transplanted into genetically altered pig embryos will migrate to the animal’s brain and alter its behavior or cognitive state” [22]. As has been discussed in this paper, there is no consensus on accurately assessing what it means to possess a human-like cognitive state or attributes of personhood. And “a being with greater sentience may also be trapped in its body and suffer greatly if enhanced awareness and understanding is not accompanied by the ability to express them” [23].

The creation of chimeras holds significant risks for animal welfare due to limited understanding of the resulting biology; for example, “chimeric pigs given a specific human growth-associated gene (Beltsville pigs) suffered multiple unforeseen symptoms including diarrhea, lethargy, skin and eye problems and constant arthritic pain. Many animals may suffer before the intended model chimera is created” [23].

Nonetheless, the reality is that chimeric experimentation has already begun, while the general public remain largely unapprised and uneducated about what is involved in these practices. Cloning practices are a good example of controversial animal experimentation that continues unabated. Polling data show “64% of Americans believe that animal cloning is morally wrong, yet there is almost no public discussion of this science and no demand for tighter regulations or governmental control over it” [24].

Consequentialist issues raised by animal cloning are the possible untoward outcomes that may result from this science, and the pain and suffering animals experience in the cloning process [24]. From a deontological perspective, the intrinsic value of animals should keep them from being objectified. There are high rates of miscarriage, stillbirth, early death, genetic abnormalities, and chronic diseases among cloned animals. Even when cloning “efficiency” rates are at their best, the majority of attempts fail. One study touting a “highly efficient” method for cloning pigs claims efficiency rates of only 5 to 12% [24]. Of the live clones born, many experience compromised health status or early death. In one study of cloned pigs, “researchers reported a 50% mortality rate for the live offspring…from ailments including chronic diarrhea, congestive heart failure, and decreased growth rate” [24]. To further highlight the objectification of animals in cloning practices, scientists no longer assign names to the cloned animals as was initially done, (e.g., Dolly, the cloned sheep in 1996); cloned animals are given numbers, just like practices of numbering livestock in factory farming. 

Biotechnology proponents will point out that when assisted reproductive technologies (ARTs) were initially introduced, there was moral outrage and push-back; over time, ART procedures have become accepted and commonplace, both in secular society and religious contexts, as well. Just give it time, proponents say, and any intuitive moral unease or disgust response will dissipate [25]. 

## 5. Nonhuman Animal Moral Status

The precautionary principle is a matter of debate in the field of ethics, with differing interpretations and thresholds applied. The risk-benefit analysis of any situation will be skewed by the perspectives being applied of stakeholders. For example, a strong or “strict interpretation of the precautionary principle would prohibit the development of chimeras with advanced mental capacities due to their potential risks” [23]. Ethical justifications through a “weak” proportionality argument would be that “the means used should not be excessive in relation to the intended goal, and that the option with the greatest balance of benefits over disadvantages should be used” [23].

While it is certainly true that we face significant organ shortages for human transplants in this country, have we tried *all* potential options to rectify the situation before experimenting on pigs? One reason cited for organ shortages in the United States is having an “opt-in” option, leaving it to the discretion of people while they are alive if they wish to be organ donors, or to surrogates when someone dies. But in many countries, laws have been implemented that presume consent to organ donation upon death, unless an objection has been registered; this presumed consent is known as "opting out”. With evidence that countries instituting presumed consent have increased organ availability [26], this author would assert that it is time for the U.S. to change from an “opt in” to an “opt out” model [27] as a first recourse to addressing organ shortages.

In revisiting the three literary examples provided at the beginning of this article, the status quo effects of pig commodification can be discerned in all three stories (e.g., the runt is left to die, the piglets are sold at market). The most significant change from one story to the next is found in the child (protagonist) challenging parental/authority pronouncements of “no choice”; these calls for honest self-reflection are necessary in a psychological process of generating alternate “solutions” to human problems. Have all options *really* been explored? Or do issues of personal pride or cultural practice serve to “limit” choices? For example, in *A Day No Pigs Would Die*, we learn that the neighbors have been successful in obtaining food, and Rob’s older siblings are married with families. These potential sources of help have not been explored. And, assuming that killing Pinky was indeed the last resort, Rob’s father could have done this without forcing him to be there too. In *Saving Marty*, we experience the animal moral status tide turning vis-à-vis Marty’s relationship with Renzo. When Renzo’s mother feels they can no longer afford to keep Marty, she does not resort to a “no choice” of selling him at auction; rather she explores options wherein Marty will be protected and have a decent quality of life (animal sanctuary or zoo). These stories teach us that “we need not accept the rules that our society or species, family or fate seem to have written for us. We can choose a new way” [13].

So, while a paradigm regarding it normal for animals to be used for human benefit is perpetuated “by powerful vested interests…(in) the role they have played in shaping the moral and political agenda” [28], and through cultural or religious traditions, we must remember that these forces are not immutable; they are “made, practiced, and applied by human beings capable of learning, and can change” [20].

As Koplin and Wilkinson (2019) point out, nobody would accept infant organ harvesting. While the infant’s cognitive capacities are not those of a fully grown human, they would develop further if they had not been killed. “If these factors do not mitigate the wrongness of killing an infant, they cannot mitigate the wrongness of killing human–pig chimeras who also would have gone on to develop morally relevant cognitive capacities” [29].

Among the reasons cited for moral status attribution is the “capacity to formulate and act in accordance with one’s own reason, understanding and deliberation recognition of one’s own interests, and by extension mutual recognition of the interests and dignity of other dignity-bearers” [23]. But as a mental health clinician with over three decades of counseling experience, I would counter that these proposed qualifying factors for animal moral worth are set at levels that most humans cannot meet. There are countless numbers of people who do not “act in accordance with one’s own reason”, as evidenced by addictive, self-sabotaging, emotionally reactive, or inconsistent behaviors. 

So, where do children’s stories and cultural narratives fit within the moral “intuition versus reasoning” divide? As fMRI studies increasingly substantiate an interplay between cognitive and affective dimensions of moral decision-making, in these stories we encounter protagonists who hold strong to moral intuitions, and challenge adult or cultural “reasoning” that does not align with their direct experience. Challenges to status quo are reflective of adolescent development, but they also serve an important function of safeguarding moral status from being dismissed, ignored, or explained away via rationalization. While we teach our children to be accepting of others and examine biases perpetuating prejudices and intolerance among human beings, these same self-reflective and compassionate behaviors should be extended to nonhuman animals, too.

## 6. Conclusions

In Charlotte’s Web, when Wilbur reflects on his friendship with Charlotte, he says, “It is not often that someone comes along who is a true friend and a good writer. Charlotte was both” [2]. This statement, although simply written for a children’s audience, is quite profound. Had Charlotte been *only* a good friend to Wilbur, it would have made their relationship special, but it would not have been enough to save his life—that feat required additional skill sets: Charlotte’s ability to analyze the cultural mores of the society in which they were living, i.e., Who holds the power, and what are the priorities of those in power? How can we simultaneously meet the needs of those in control while protecting Wilbur from harm? The next essential skill set Charlotte utilizes is finesse of communication – the ability to find, and write, words that will grab the attention of society-at-large. This is no easy task. There is a lot of noise in the media, with headlines centered on the loudest noisemakers, not necessarily the most important issues. In a world where rights violations abound, where environmental and animal rights activists sometimes feel compelled to resort to “radical” or disruptive means, there are others who can also give voice. Ethicists, whether at the patient’s bedside, the ethics committee, or the animal rights policy meeting, are devoted to the protection of all living beings too.

The three stories chosen for this paper reflect how, over the greater part of this past century, the Western literary world has penned a moral paradigm shift—from viewing animals as property, to viewing them as rights bearers worthy of dignity and protection. These children’s stories, written by adult authors, serve not only to reflect societal norms but to question them. They challenge the notion of “ready-made ethical systems from outside or above…by encouraging children to shape personal ethics” [16]. And they challenge the adults who have grown up reading these stories, or those writing the next ones, to make the changes necessary for a more just, compassionate society for all.
